# Exploring Glycocalyx shedding markers: A comparative analysis of plasma markers among premenopausal and postmenopausal women

**DOI:** 10.14814/phy2.70964

**Published:** 2026-06-23

**Authors:** Mounica Ayyalasomayajula, David N. Proctor, Janhavi J. Damani, Christine Bowlus, Jigar Gosalia, Jocelyn M. Spicuzza

**Affiliations:** ^1^ Department of Kinesiology The Pennsylvania State University University Park Pennsylvania USA; ^2^ Integrative and Biomedical Physiology, Huck Institutes of the Life Sciences The Pennsylvania State University University Park Pennsylvania USA; ^3^ Department of Nutritional Sciences The Pennsylvania State University University Park Pennsylvania USA; ^4^ Department of Psychiatry University of Pittsburgh School or Medicine Pittsburgh Pennsylvania USA

**Keywords:** endothelial glycocalyx, HS, menopause, Sydecan‐1

## Abstract

The endothelial glycocalyx (eGC) is a protein‐rich, hairlike structure covering the luminal surface of endothelial cells, vital for vascular health. Aging and cardiovascular disease (CVD) can compromise eGC integrity, potentially preceding conventional indicators of vascular dysfunction such as reduced endothelium‐dependent vasodilation and increased arterial stiffness. SDC‐1 and HS are key glycosaminoglycans and validated plasma biomarkers reflecting glycocalyx integrity. During menopause the decline in estrogen, a vascular‐protective hormone, has been linked to increased glycocalyx degradation and decreased nitric oxide (NO) synthesis contributing to endothelial dysfunction. This study examined glycocalyx degradation in premenopausal females versus postmenopausal females (early and late stages). Resting plasma samples from premenopausal (*n* = 11), early‐postmenopausal (*n* = 12) and late‐postmenopausal women (*n* = 13) were analyzed via ELISA for SDC‐1 and HS. Late postmenopausal females had significantly higher mean plasma HS concentrations compared to early postmenopausal and premenopausal groups (4049.9 ± 1595.4 vs. 3919.0 ± 999.2, 2986.4 ± 1493.7 ng/mL, *p* = 0.003). Conversely, SDC‐1 levels were higher in premenopausal women (*p* = 0.036). No correlations were observed between these biomarkers and flow‐mediated dilation (all, *p* > 0.05). Overall, results suggest eGC integrity varies across reproductive stages, emphasizing the need for further research into mechanisms and interventions to preserve vascular health in aging females.

## 1 INTRODUCTION

Cardiovascular disease (CVD) remains the leading cause of morbidity and mortality worldwide for both men and women (Tsao et al., 2022). Although men generally exhibit higher CVD prevalence than age‐matched premenopausal women, this disparity narrows significantly following menopause, where CVD prevalence is higher in postmenopausal women compared to age‐matched men (Celermajer et al., 1994; Kannel et al., 1976; Reckelhoff & Fortepiani, 2004). While the link between CVD progression and postmenopause is extensively documented, changes occurring during perimenopause remain less understood, despite this phase representing a critical therapeutic window (Cho et al., 2020; El Khoudary et al., 2020; Khan et al., 2018). The menopause transition is associated with a higher prevalence of CVD risk factors (Ryczkowska et al., 2022), underscoring the need for targeted intervention during the menopausal transition to mitigate CVD risk in later postmenopausal years. Endothelial dysfunction is an early prognostic indicator of atherosclerosis. Following menopause, the progressive decline in endogenous estradiol induces adverse changes in vascular health, which is a primary contributor to menopause‐mediated endothelial dysfunction (Lv et al., 2023; Moreau, 2018; Somani et al., 2019). These changes include a measurable decline in endothelium‐dependent vasodilation, often assessed noninvasively by flow‐mediated dilation (FMD) (Thijssen et al., 2019), and a concomitant increase in arterial stiffness (Ogola et al., 2018; Weng et al., 2013). While endothelial function is stable in premenopausal women across the reproductive years (Shenouda et al., 2018; Williams et al., 2024), resting FMD has been shown to progressively decline across the peri‐ and postmenopausal years, with the lowest FMD seen among late‐postmenopausal women (Celermajer et al., 1994; Delgado Spicuzza et al., 2023; Delgado Spicuzza, Gosalia, Studinski, et al., 2024; Delgado Spicuzza, Gosalia, Zhong, et al., 2024; Holder et al., 2021; Moreau et al., 2012; Somani et al., 2022; Wenner et al., 2024). This evidence suggests that menopause‐induced hypoestrogenism negatively impacts endothelial function.

One particular target of interest to improve endothelial function is the endothelial glycocalyx (eGC), a complex luminal structure, composed primarily of proteoglycans like SDC‐1 and glycosaminoglycans such as HS that are essential for vascular homeostasis. The eGC has several functions such as a mechanotransducer of hemodynamic shear stress to regulate nitric oxide synthesis and release, modulator of vascular permeability, and limits cell adhesion (Aldecoa et al., 2020; Haymet et al., 2021; Machin et al., 2019). eGC degradation occurs with various diseases and leads to the shedding of eGC components into systemic circulation (Cao et al., 2019). Consequently, circulating levels of SDC‐1 and HS have emerged as key biomarkers for assessing endothelial injury and glycocalyx degradation severity in conditions such as surgical trauma and hemorrhagic shock (Becker et al., 2015; Passov et al., 2021; Rehm et al., 2007). Estradiol is known to protect the eGC from degrading; therefore, the loss of estrogen during menopause may accelerate eGC deterioration, ultimately contributing to menopause‐mediated endothelial dysfunction (Bartosch et al., 2017; Potje et al., 2023).

Glycocalyx degradation has been shown to be implicated by chronological aging and may precede the onset of overt endothelial dysfunction, such as occurs in late‐postmenopause (Machin et al., 2019). However, the impact of reproductive aging on glycocalyx composition across female reproductive stages remains limited, representing a critical gap in understanding how menopause mediates endothelial glycocalyx degradation and subsequent endothelial function. The purpose of this study was to examine the profiles of glycocalyx shedding markers in estrogen‐replete premenopausal females and hypoestrogenic postmenopausal females (both early‐ [1–6 years following the final menstrual period (FMP)]) and late‐postmenopausal stages (6+ years since the FMP). We hypothesized that chronic hypoestrogenism in late‐postmenopause would coincide with greater endothelial glycocalyx degradation, as measured by greater plasma concentrations of SDC‐1 and HS compared to estrogen‐replete premenopausal women.

## 2 MATERIALS AND METHODS

### 2.1 Ethical approval

This study was approved by the Pennsylvania State University Institutional Review Board (IRB# STUDY00010017) and conducted in accordance with the Declaration of Helsinki. All participants provided written informed consent. The study was part of a larger registered clinical trial (NCT03644472) investigating the influence of menopausal stage on vascular endothelial function, with related findings previously published (Delgado Spicuzza et al., 2023; Delgado Spicuzza, Gosalia, Studinski, et al., 2024; Delgado Spicuzza, Gosalia, Zhong, et al., 2024).

### 2.2 Participant characteristics

This cross‐sectional study included premenopausal women (*n* = 11) with regular menstrual cycles or using hormonal contraception and postmenopausal women classified as either early (*n* = 12, 1–6 years since final menstrual period) or late (*n* = 13, >6 years post final menstrual period). Menopause status was defined using the Stages of Reproductive Aging Workshop (STRAW+10) criteria (Harlow et al., 2012). Menstrual cycle was not controlled for in premenopausal women. Eligible participants did not have overt chronic disease as confirmed by a physician reviewed medical history questionnaire and venous blood chemistry (hematological, liver, and kidney function), and met the following criteria: resting brachial blood pressure <130/80 mmHg, body mass index between 18.5 and 35 kg/m^2^, fasting plasma glucose <100 mg/dL or HbA1c <6.0%, fasting plasma low‐density lipoprotein <160 mg/dL, nonsmoker, not taking any cardiovascular medications or hormone replacement therapy (postmenopausal group only), and had not donated blood or blood products in the past 3 months. Physical activity was determined using the self‐reported International Physical Activity Questionnaire (Booth, 2000).

### 2.3 Blood sampling and storage

Venous blood samples were collected from the antecubital vein in 6 mL sodium heparin tubes (BD Vacutainer, Franklin Lakes, N.J., USA), then immediately centrifuged at 3000 rpm (3000 g) at $-4^{{}^{\circ}}C$ for 4 min. The plasma was then aliquoted and stored at $-80^{{}^{\circ}}C$ for later analysis.

### 2.4 ELISA analysis

Plasma concentrations of SDC‐1 (SDC‐1) and HS (HS) were quantified in duplicate from all participants using commercial Enzyme‐Linked Immunosorbent Assay (ELISA) kits.

Plasma SDC‐1 concentrations were determined using a solid‐phase sandwich ELISA kit (Diaclone SAS, catalogue no: 950.640.096, Besancon, France) as previously described (Hulde et al., 2021). The assay sensitivity was 4.94 ng/mL, and the intraassay coefficient of variation (CV) for duplicates was <10%.

Plasma HS concentrations were measured using a competitive inhibition enzyme immunoassay (Cusabio, catalogue no: CSB E09585h, USA) following established protocols (Hulde et al., 2018, 2021). After a 1:2 sample dilution, HS in the sample competed for binding sites with HS precoated on the microplate. This assay had a minimum detectable dose of <31.25 ng/mL, and the intraassay CV was <20%.

### 2.5 Flow‐mediated dilation (FMD)

Flow‐mediated dilation (FMD) of the brachial artery, which primarily reflects nitric oxide (NO)‐mediated endothelium‐dependent vasodilation, was assessed in the right arm, positioned at 80 to 90 degrees from the torso. A rapid inflation/deflation pneumatic cuff (Hokanson) was placed around the forearm just below the elbow (distal to the olecranon process). Longitudinal B‐mode images of the brachial artery in the distal upper arm were captured using a multifrequency linear array probe connected to a high‐resolution ultrasound machine (Phillips IU22). Doppler velocity was simultaneously recorded at a 60‐degree insonation angle, with the sample volume adjusted to match the vessel size. Resting brachial artery diameter and blood velocity were recorded for 1 min. The pneumatic cuff was then inflated to 250 mmHg for 5 min, during which arterial lumen diameter and blood velocity were continuously measured. After cuff deflation, imaging continued for an additional 3 min. To ensure consistency between measurements, the transducer placement was marked on the participant's arm. All FMD tests were conducted by the same sonographer, who demonstrated a coefficient of variation of 1% for baseline diameter and 16.9% for relative FMD, aligning with expert recommendations (Thijssen et al., 2019).

### 2.6 Arterial stiffness measurements

Ankle‐brachial pulse wave velocity (PWV) was measured by placing four blood pressure cuffs securely around the participant's upper arms and ankles; ECG electrodes were placed on the inner right and left wrists, and the phonocardiogram sensor was placed on the rib cage location according to manufacturer instructions (Colin VP). The automatic measurement was initiated and lasted between 45 s and 1 min. PWV and ankle‐brachial index (ABI) were measured supine in triplicate separated by 1 min.

### 2.7 Statistical analysis

The parent study (Delgado Spicuzza et al., 2023; Delgado Spicuzza, Gosalia, Studinski, et al., 2024; Delgado Spicuzza, Gosalia, Zhong, et al., 2024) was designed to achieve 80% power for detecting a clinically significant between‐group difference in endothelial function before and after IR injury the primary outcome, with a 2‐sided type 1 error rate of 5%. In this ancillary study, glycocalyx degradation was assessed as an exploratory outcome, and thus, no formal power calculation was performed. Descriptive statistics are reported as mean ± standard deviation (SD), with ranges provided when appropriate. There were no outliers in the glycocalyx data as assessed by ± 3 SD. For statistical analysis, the average values of the sample duplicates were used. If data followed a normal distribution, between‐group differences were tested using parametric models, such as independent sample *t*‐test and one‐way ANOVA. However, data that followed a nonnormal distribution were log‐transformed prior to parametric testing. If transformations were unsuccessful at normalizing data distribution, nonparametric tests such as Mann–Whitney *U* tests and Kruskal–Wallis tests were used. Multiple comparisons were corrected with appropriate post hoc tests, such as the Tukey–Kramer for ANOVA or Bonferroni test for the Kruskal–Wallis. FMD was calculated as the percent increase from baseline to peak diameter during reactive hyperemia ((peak diameter‐baseline diameter)/baseline diameter × 100%). Allometrically scaled FMD (adjusted FMD) was calculated as *ln*(peak diameter)—*ln*(baseline diameter) (Atkinson, 2014; Atkinson & Batterham, 2013). Assumptions underlying allometric scaling were evaluated by assessing the log–log linear relationship between baseline and peak artery diameter and by examining the independence of adjusted FMD from ln(baseline diameter) using linear regression analyses. When a main effect for treatment or menopausal stage by treatment existed, the conservative Bonferroni correction method was used to adjust for multiple comparisons for all outcomes (i.e., unadjusted FMD, vascular hemodynamics, resting blood pressure), however as recommended per Atkinson and colleagues, the Fisher's Least Significant Difference correction method was used to adjust for multiple comparisons for adjusted FMD data only (Atkinson & Batterham, 2013). All statistical analyses were conducted using IBM SPSS Statistics version 29.0.2.0 (Armonk, NY; IBM Corp). A significance level of *p* < 0.05 was used for all analyses.

## 3 RESULTS

### 3.1 Participant characteristics

Among the 11 premenopausal participants, six were using hormonal contraception (three with Mirena IUD, one with Nexplanon, and one Camila). Of the five premenopausal females not using hormonal contraceptives, four reported having regular menstrual cycles. The time since menopause for early (*n* = 12) and late postmenopausal females (*n* = 13) averaged 4 ± 1.6 years and 15 ± 5.5 years, respectively, with a significant difference between the two groups (*p* < 0.001). Females in the late postmenopausal group were significantly older (63 ± 5 years) compared to those in the early postmenopausal group (56 ± 3 years, *p* < 0.001). BMI did not differ significantly between the groups (*p* = 0.34); however, total cholesterol (*p* = 0.002) and LDL concentrations (*p* = 0.03) were significantly lower in premenopausal females compared to the postmenopausal groups (Table 1).

**TABLE 1:** Participant characteristics.

Variable	Premenopausal	Early postmenopausal	Late postmenopausal	*p* Value
*n*	11	12	13	—
Age (year)	25±3 ^b,c^	56±4 ^a,c^	64±5 ^a,b^	<0.001
Years since menopause	N/A	4±2 ^c^	14±5	<0.001
BMI (kg/m^2^)	25±3 ^c^	24±3	23±3 ^a^	0.34
Weight (kg)	72±12 ^c^	67±11	59±5 ^a^	0.01
Height (cm)	168±6 ^c^	167±6	162±4 ^a^	0.01
Resting systolic BP (mmHg)	107±8	112±11	115±11	0.22
Resting diastolic BP (mmHg)	70±7	67±9	65±6	0.26
Resting HR (beats/min)	66±13	62±6	65±13	0.66
eGFR (mL min^−1^/1.73m^2^)	104±11 ^b,c^	82±12 ^a^	84±13 ^a^	0.02
Total cholesterol (mg/dL)	165±34 ^b,c^	210±32	215±30 ^a^	0.002
LDL (mg/dL)	88±31 ^b,c^	123±31 ^a^	117±30 ^a^	0.03
HDL (mg/dL)	66±9 ^b,c^	65±14 ^c^	80±17 ^a,b^	0.03
Triglycerides (mg/dL)	66±30	84±30	83±33	0.32
Fasting glucose (mg/dL)	87±5	89±5	92±8	0.22
Hematocrit (%)	42±3	41±3	40±3	0.61
Hemoglobin (g/dL)	14±1	13±1	14±1	0.48
Physical activity (MET‐week)	3945±3069	3064±3822	2538±1745	0.55
Parturition number	0	2±1	2±1	0.37
Pulse wave velocity (m/s)	10.72±1.06	12.76±1.92 ^a^	14.34±2.92 ^a^	<0.001
Brachial Ankle index	1.06±0.11 ^b,c^	1.12±0.04	1.12±0.09 ^a^	0.006
Baseline diameter (mm)	3.4±0.2	3.5±0.5	3.3±0.4	0.002
FMD (%)	7.8±2 ^a^	7.1±3	4.5±2	0.005
Adjusted FMD (%)	7.7±2 ^a^	7.0±2 ^a^	4.5±2	0.005
Shear rate AUC (10^−3^)	17.9±5	23.1±7	23.8±6	0.112
Baseline blood flow (mL/min)	35±24	37±24	57±24	0.062
Peak blood flow (mL/min)	356±156	483±156	415±156	0.053
Baseline velocity (m/s)	7±4	9±4	14±4	0.593
Peak velocity (m/s)	69±23	84±23	94±23	0.99

*Note*: Data presented as mean ± SD. *p* values reflect overall group differences (one‐way ANOVA). Post hoc pairwise differences were identified with Tukey's test and are indicated by superscripts as follows: ^a^ *p* < 0.05 versus premenopausal; ^b^ *p* < 0.05 versus early postmenopausal; ^c^ *p* < 0.05 versus late postmenopausal.

Abbreviations: AUC, area under the curve; BP, blood pressure; eGFR, estimated glomerular filtration rate; FMD, flow‐mediated dilation; HR, heart rate.

### 3.2 Endothelial Glycocalyx metabolites

**FIGURE 1:**
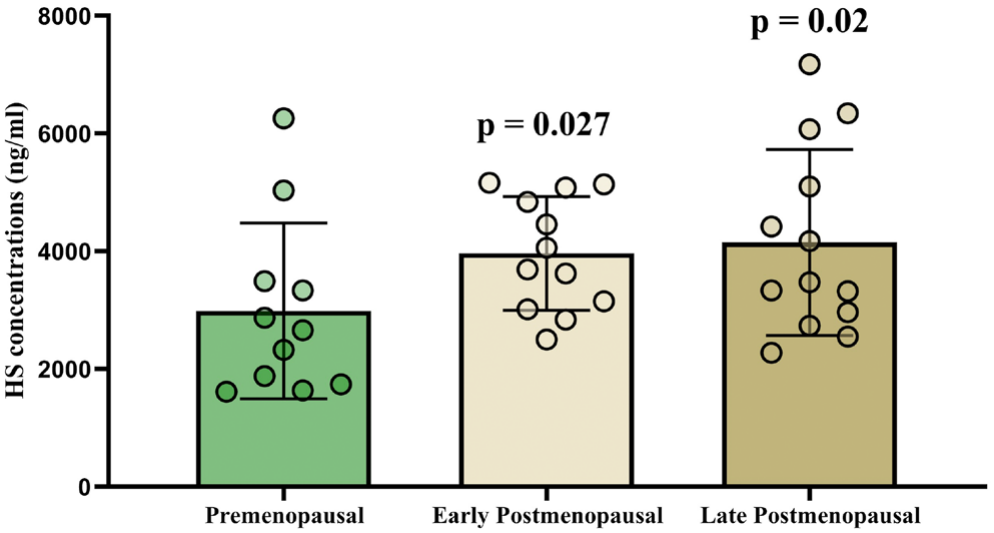
Comparison of mean HS (HS) concentrations between premenopausal and postmenopausal women using an independent *t*‐test. Postmenopausal women exhibited significantly higher HS levels compared to premenopausal women.

**FIGURE 2:**
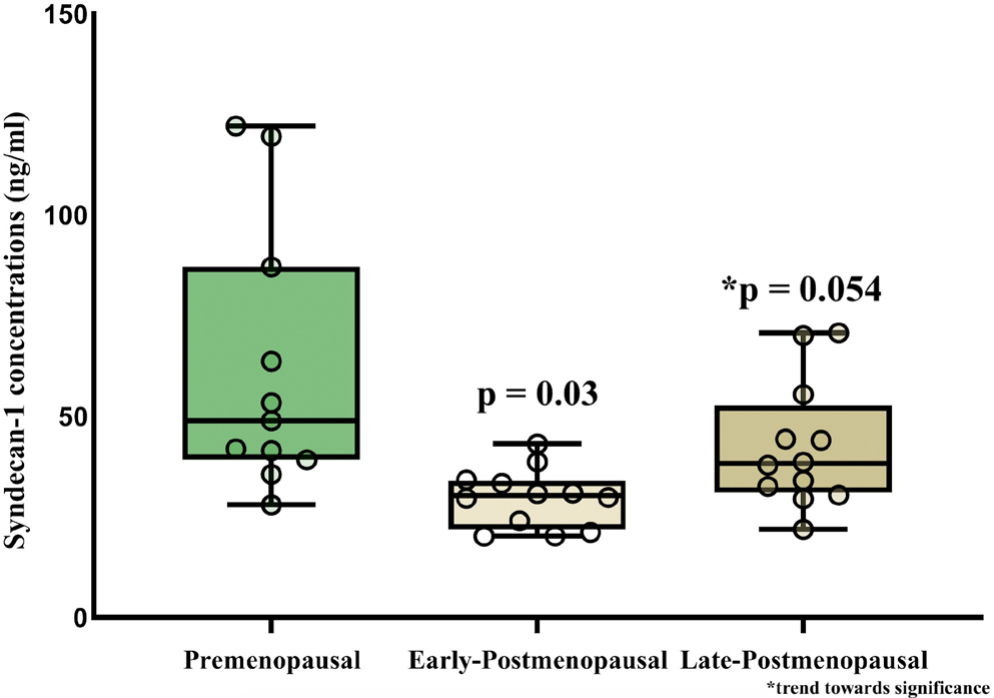
SDC‐1 concentrations across premenopausal, early postmenopausal, and late postmenopausal women were analyzed using the Kruskal–Wallis test. Early postmenopausal women showed significantly lower SDC‐1 levels compared to premenopausal women, while the difference between early postmenopausal and late postmenopausal women is following a trend towards significance. Bonferroni test was used for post hoc comparisons.

In line with our hypothesis, postmenopausal females (*n* = 25, 3732.6 ± 238.2 ng/mL) exhibited higher mean plasma concentrations of HS than premenopausal females (*n* = 11, 2986.4 ± 1493.7 ng/mL), with a large effect size (Cohen's d=0.9) (Figure 1). In contrast to our hypothesis, premenopausal females (*n* = 11, 61.8 ± 33.1 ng/mL) displayed higher median concentrations of SDC‐1 compared to postmenopausal females (*n* = 25, 40.8 ± 27.4 ng/mL) (Figure 2). For SDC‐1 concentrations, the Kruskal–Wallis test yielded a substantial effect size ($\eta^{2}=0.29$), and the Mann–Whitney *U* test indicated a large effect (Cohen's r=0.41) (Table 2).

**TABLE 2:** HS and SDC‐1 concentrations.

Parameter	Groups		
	Premenopausal	Early postmenopausal	Late postmenopausal
**SDC‐1**			
*n*	11	12	13
Mean ± SD	61.8 ± 33.2	29.6 ± 7.3	42.4 ± 15.6
Median	48.9	30.28	38.2
IQR	47.9	12.1	21.7
95% CI	39.6–84.2	25.04–34.27	32.5–52.3
**HS**			
*n*	11	12	13
Mean ± SD	2986.4 ± 1493.7	3919 ± 999.2	4049.9 ± 1595.4
Median	2660.4	3692.15	3474.2
IQR	1750.7	2068.00	2365.6
95% CI	1982.9–3989.9	3247.8–4590.3	2978–5121.6

#### 3.2.1 Heparan sulfate concentrations

**FIGURE 3:**
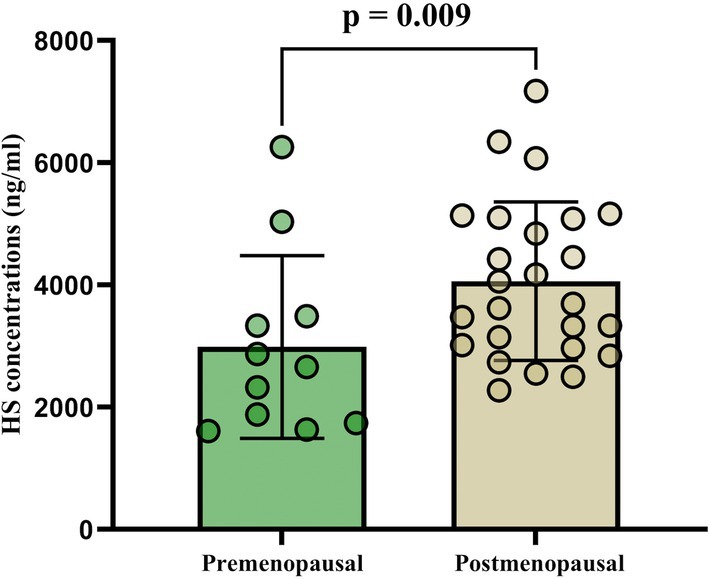
Comparison of median SDC‐1 concentrations between premenopausal and postmenopausal women using the Mann–Whitney *U* test. SDC‐1 levels are significantly lower in postmenopausal women compared to premenopausal women.

When categorized by pre‐ and postmenopausal stages, early‐postmenopausal (3919 ± 999.2 ng/mL, p=0.027) and late‐postmenopausal females (4049.9 ± 1595.4 ng/mL, p=0.027) had significantly higher HS concentrations than premenopausal females (2986.4 ± 1493.7 ng/mL) (Figure 3). ANOVA indicated a large effect size between groups ($\eta^{2}=0.18$), further supporting these differences.

#### 3.2.2 Syndecan‐1 concentrations

**FIGURE 4:**
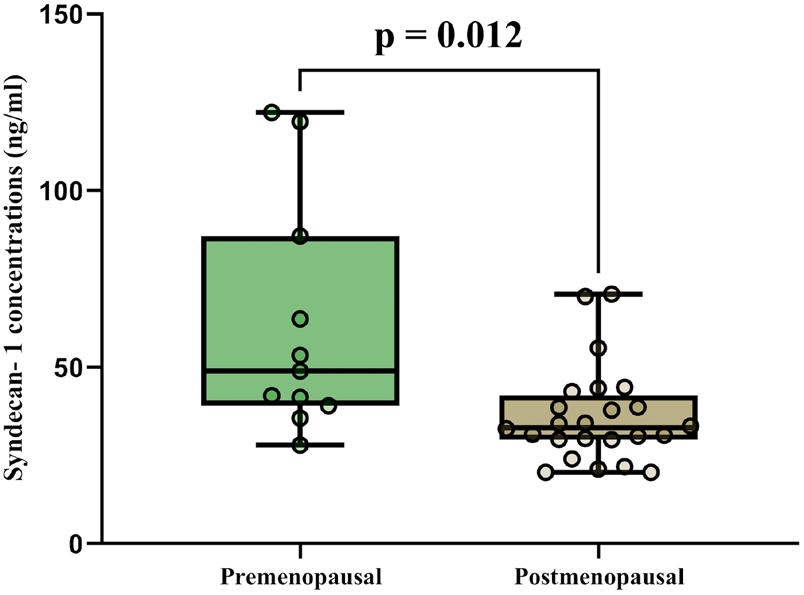
Comparison of HS (HS) concentrations across premenopausal, early postmenopausal, and late postmenopausal women using ANOVA. There is a significant difference in HS concentrations between the groups, with both early and late postmenopausal women showing higher levels compared to premenopausal women (premenopausal with earlypostmenopausal, premenopausal with latepostmenopausal. Tukey test was used for Post hoc comparisons).

Median plasma SDC‐1 concentrations were 48.9 ng/mL [95% CI: 39.5–84] in premenopausal females, 30.3 ng/mL [95% CI: 25–34.2] in early postmenopausal females, and 38.2 ng/mL [95% CI: 32.5–52.3] in late postmenopausal females (Figure 4). These differences suggest meaningful variations by menopausal status, as indicated by the Kruskal–Wallis test effect size ($\eta^{2}=0.29$).

### 3.3 FMD and PWV versus eGC metabolites

Linear regression analyses adjusting for $\ln\left(\text{baseline diameter}\right)$ and menopausal group demonstrated that HS was not associated with FMD (B=−0.177, SE=2.930, p=0.952) or adjusted FMD (B=2.851, SE=2.846, p=0.325). Similarly, Syndecan‐1 (SDC‐1) was not associated with FMD (B=−0.007, SE=0.016, p=0.654) or adjusted FMD (B=−0.018, SE=0.015, p=0.238). Across all models, late postmenopausal women demonstrated significantly lower FMD and adjusted FMD compared with premenopausal women (FMD‐HS model: B=−3.379, SE=1.175, p=0.008; adjusted FMD‐HS model: B=−3.675, SE=1.108, p=0.003; FMD‐SDC‐1 model: B=−3.415, SE=1.100, p=0.004; adjusted FMD‐SDC‐1 model: B=−2.882, SE=1.028, p=0.009), whereas early postmenopausal women were not significantly different from premenopausal women. $\ln\left(\text{baseline diameter}\right)$ was not a significant predictor in any model (all p>0.05). No significant correlations were found between FMD and HS (p=0.813, r=−0.044) or FMD and SDC‐1 (p=0.952, $\rho \left(\mathrm{rho}\right)=-0.011$). In contrast, a significant positive correlation was found between brachial‐ankle PWV and HS in early postmenopausal women (p=0.033, r=0.642).

## 4 DISCUSSION

This study investigated the integrity of the endothelial glycocalyx (eGC) across postmenopausal stages by measuring plasma concentrations of two key glycocalyx degradation biomarkers, SDC‐1 and HS. We hypothesized that menopause‐induced hypoestrogenism would be associated with greater eGC degradation, reflected by elevated levels of both biomarkers compared to young premenopausal women. Our secondary hypothesis was that FMD would be correlated with the plasma concentrations of SDC‐1 and HS. Our findings, however, revealed a more complex and nuanced picture. While HS plasma concentrations were indeed higher in postmenopausal women, supporting our initial hypothesis, plasma concentrations of SDC‐1 were higher in the premenopausal group. These divergent patterns suggest that menopause‐mediated hypoestrogenism may induce a fundamental shift in eGC composition.

A growing body of evidence suggests CVD is linked to the health of the endothelial glycocalyx (eGC) (Kim et al., 2017; Machin et al., 2019). Plasma biomarkers of eGC degradation, such as HS and SDC‐1, correlate well with the severity of endothelial dysfunction, a known antecedent to CVD development (Cao et al., 2019; Kim et al., 2017; Kršek et al., 2024). During the female menstrual cycle, the moderate, sex hormone‐induced shedding of eGC appears to be a naturally well‐tolerated byproduct of adaptive mechanisms to preserve endothelial function (Hulde et al., 2018; Mensah et al., 2017). Healthy young women demonstrate a remarkable capacity for eGC regeneration after shedding, which is attributable to favorable oxidative status and higher bioavailability of nitric oxide (NO) (Hulde et al., 2018; Mensah et al., 2017). Prior studies have shown that median SDC‐1 concentrations are highest during the luteal phase (12.6 and 12.6 ng/mL, respectively) and lowest during the follicular phase (11.7 and 11.3 ng/mL, respectively) in young, healthy premenopausal women (Hulde et al., 2018, 2021). The authors attributed these small, yet significant, variations in circulating SDC‐1 across the menstrual cycle to fluctuating levels of female sex hormones and reduced estrogen‐mediated inhibition of eGC sheddase activity, more specifically matrix metalloproteinases (MMPs) (Potje et al., 2023). In contrast to our hypothesis, average concentrations of SDC‐1 were lower in our postmenopausal groups (42 ng/mL) than in our premenopausal group (62 ng/mL). While we have no single clear explanation, this paradoxical result may not indicate greater vascular dysfunction in premenopausal women but may instead reflect a shift from a state of dynamic physiological remodeling to one of chronic eGC degradation following menopause. Higher SDC‐1 concentrations in premenopausal women may be a signature of a healthy, hormonally driven turnover, where cyclical shedding is balanced by robust regeneration. However, whether menopause‐induced hypoestrogenism has a similar effect on eGC degradation in postmenopausal women has not been explored previously.

The present measurements of SDC‐1 and HS appear to be the first eGC data available for healthy postmenopausal women (Table 2). To date, only one study has reported a median plasma SDC‐1 concentration of 21.4 ng/mL in healthy midlife women (*n* = 35, 47.4 ± 5.5 year) (Table A1). However, this study did not control for time since menopause (i.e., postmenopausal stages) (Nam et al., 2021). Stratifying postmenopausal women into early‐ and late‐postmenopausal stages may partly explain the higher median plasma concentrations observed in this study (Early‐PM: 30.3 ng/mL; 95% CI = 25.0–34.3; Late‐PM: 38.2 ng/mL; 95% CI = 32.5–52.3) (Delgado Spicuzza et al., 2023; Delgado Spicuzza, Gosalia, Studinski, et al., 2024; Delgado Spicuzza, Gosalia, Zhong, et al., 2024) compared to values observed in Nam et al. (2021). Additionally, the strict control over CVD risk factors and medication use in the present sample of women may explain the lack of a significant difference in SDC‐1 and HS concentrations between early‐ and late‐postmenopausal groups. Together, these findings establish a foundation for examining how menopausal stage related differences in eGC components may translate to alterations in vascular function.

### 4.1 eGC degradation and macrovascular endothelial function

A degraded eGC is less effective at mechanotransduction, leading to impaired NO synthesis and a blunted vasodilatory response. Contrary to our hypothesis, we did not observe significant correlations between plasma eGC concentrations and flow‐mediated dilation values in either pre‐ or postmenopausal groups (overall *r* = −0.044, *p* > 0.05). All participants were normotensive and metabolically healthy, likely exhibiting low inflammation and oxidative stress. While this may not fully explain the wide variability in eGC concentrations observed in this study, the selective inclusion criteria likely limited variability in FMD. The lack of association may reflect the fact that glycocalyx degradation precedes other vascular aging markers such as endothelial function (Machin et al., 2019). Compensatory mechanisms, including loss of specific eGC components like SDC‐1 or HS, and alternative endothelium‐independent vasodilatory pathways may offset menopause‐related declines in endothelial function (Wu et al., 2018). These factors could explain the absence of correlation between eGC and FMD, as well as preserved endothelial function despite elevated eGC biomarkers reported elsewhere (Gimblet et al., 2024). Lastly, circulating eGC metabolites may derive from multiple tissues rich in glycosaminoglycans, such as the liver and kidneys, complicating interpretation (Hahn et al., 2021). While glycocalyx degradation has been shown to primarily disrupt microvascular function, our assessments were limited to measuring macrovascular function, which may not fully reflect microvascular eGC changes (Jaarsma et al., 2017; Machin et al., 2019; Salmon & Satchell, 2012; Yamaoka‐Tojo, 2020). In the present study, neither HS nor Syndecan‐1 (SDC‐1) were independently associated with FMD or adjusted FMD after accounting for menopausal status and baseline artery diameter. These findings suggest that circulating glycocalyx degradation markers may not directly reflect conduit artery endothelial function in healthy women across menopausal stages. In contrast, menopausal status appeared to be a stronger determinant of vascular function, as late postmenopausal women consistently demonstrated lower FMD and adjusted FMD compared with premenopausal women across all regression models. This observation is consistent with the well‐established decline in endothelial function following menopause, likely driven by reduced estrogen availability, impaired nitric oxide bioavailability, and increased vascular oxidative stress (Moreau et al., 2012, 2020). Interestingly, early postmenopausal women were not significantly different from premenopausal women, which may suggest that endothelial dysfunction becomes more pronounced later in the postmenopausal transition. Additionally, ln(baseline diameter) was not a significant predictor in any model, indicating that the observed group differences were not explained by differences in vessel size. Collectively, these findings suggest that reproductive aging negatively impacts endothelial function; however, whether circulating glycocalyx shedding markers are predictive of endothelial function requires further investigation. Although we did not observe any correlations with FMD in our study, we did identify a positive association between brachial‐ankle PWV and HS concentrations in early postmenopausal women. This finding suggests that glycocalyx degradation is more closely linked to structural alterations in the arterial wall, which may not be as readily captured by functional measures such as FMD. Overall, these findings suggest that circulating eGC biomarkers may not directly mirror macrovascular endothelial function in healthy women, highlighting the need for integrated assessments that include both microvascular function and systemic biomarkers.

### 4.2 Experimental limitations and recommendations for future studies

This study's primary strength is the novel investigation into eGC degradation across menopausal stages by utilizing the STRAW+10 criteria, in healthy women. Additionally, strict participant exclusion criteria minimized confounding health variables for a more rigorous analysis. Several limitations must also be acknowledged. Female sex hormones were not measured. Given the impact of estrogen and progesterone on vascular function, failing to account for menstrual cycle phase may have influenced the observed results. The inclusion of both oral contraceptive users and nonusers in the premenopausal group is another potential limitation. Given their widespread use, the influence of oral contraceptives on eGC metabolites should be systematically investigated in future studies. Additionally, the relatively small sample size and strict inclusion criteria in this study limit the generalizability of the findings to the broader postmenopausal population. Our study design was also cross‐sectional, precluding the assessment of longitudinal changes. Future research should implement longitudinal designs with multiple time‐point assessments and measures of female sex hormones and ovarian aging and assess other parameters that may influence eGC shedding, such as markers of inflammation and oxidative stress.

## 5 CONCLUSION

In conclusion, the present study provides novel and important data on the state of the endothelial glycocalyx in healthy pre‐ and postmenopausal women. Specifically, our findings show that premenopausal women exhibit higher mean plasma concentrations of SDC‐1, which may be a signature of a healthy, hormonally driven turnover, where cyclical shedding is balanced by robust regeneration. On the other hand, postmenopausal women show elevated plasma HS, likely because of the effects of hypoestrogenism on increasing oxidative stress and inflammation, which also contribute to eGC degradation. These divergent patterns of circulating HS and SDC‐1 underscore the profound impact of menopause on the vascular endothelium. These data highlight that the endothelial glycocalyx is sensitive to variations in female sex hormones.

## AUTHOR CONTRIBUTIONS

**Mounica Ayyalasomayajula:** Conceptualization; data curation; formal analysis; investigation; methodology; project administration; software; validation; visualization. **David N. Proctor:** Conceptualization; funding acquisition; methodology; supervision; validation; visualization. **Janhavi Damani:** Conceptualization; data curation; formal analysis; investigation; project administration. **Christine Bowlus:** Data curation; formal analysis. **Jigar Gosalia:** Data curation; formal analysis; software. **Jocelyn M. Spicuzza:** Conceptualization; funding acquisition; project administration; supervision.

## FUNDING INFORMATION

The author(s) declare financial support was received for the research, authorship, and/or publication of this article. This research was supported by the National Center for Advancing Translational Sciences, National Institutes of Health (NIH) through grant UL1TR002014. The content is solely the responsibility of the authors and does not necessarily represent the official views of the NIH. The project described was also supported by the Institutional National Research Service Award (T32) T32GM108563, T32DK120509, 5T32HL007560, and the Huck Endowment for Nutritional Research in Family and Community Medicine at Penn State College of Medicine and University Park.

## CONFLICT OF INTEREST STATEMENT

The authors declare no conflicts of interest.

## ETHICS STATEMENT

The authors have disclosed all conflicts of interest and are committed to transparency in data management.

## Data Availability

The original contributions presented in the study are included in the article/supplementary material; further inquiries can be directed to the corresponding author.

## APPENDIX 1

**TABLE A1:** Literature values for Syndecan–1 and HS.

Study	Cohort	Syndecan–1 (ng/mL)	HS (ng/mL)
**Healthy controls (Literature)**			
Steppan et al. (2011)	Healthy mixed sex	20.5 ± 5.05^b^	1960 ± 1.21^b^
Nelson et al. (2008)	Healthy mixed sex	26.0 (19–48)	Not reported^a^
Nam et al. (2021)	Healthy young females	51.1 (28.4–70.2)^b^	Not reported
Nam et al. (2021)	Healthy females >40 years	21.4 (17.0–38.2)^b^	Not reported^a^
Hofmann‐Kiefer et al. (2013)	Healthy young females	66.4 (50.7–69.7)^b^	6200 (5400–8900)^b^
Hulde et al. (2018)	Healthy premenopausal	Ovulation: 11.1 ± 2.4^b^	663 ± 35^b^
		Mid‐luteal: 12.6 ± 2.3^b^	782 ± 55^b^
Hulde et al. (2021)	Healthy premenopausal	Follicular: 11.27 (4.54–19.49)^b^	659.17 (507.89–817.94) ^b^
		Ovulation: 10.69 (4.01–20.70)^b^	620.84 (491.89–730.57)^b^
		Luteal: 12.60 (6.11–19.13)^b^	751.24 (573.76–937.89)^b^
Gimblet et al. (2024)	Healthy mixed sex	2.91 (1.824–12.581)^b^	2980 ± 871^b^
Rehm et al. (2007)	Healthy mixed sex	Not reported^a^	5590 (4820–7940)
**Pathological conditions (Literature)**			
Beurskens et al. (2020)	Sepsis (Mixed sex)	181.4 (56.6–313.2)^b^	Not reported
Kusuzawa et al. (2021)	Hemodialysis (Mixed sex)	124.6 ± 107.8 (Before hemodialysis)^b^	Not reported^a^
		229.0 ± 138.1 (After hemodialysis)^b^	
Miranda et al. (2016)	Acute coronary syndrome (Mixed sex)	62 (42–127)^b^	Not reported^a^
Rehm et al. (2007)	Major vascular surgery (Mixed sex)	12 (7.2–14.2)^b^	5900 (4530–8410)^b^
Steppan et al. (2011)	Sepsis (Mixed sex)	160 ± 109^b^	3230 ± 2430^b^
Steppan et al. (2011)	Major abdominal surgery (Mixed sex)	50.5 ± 46.9^b^	7960 ± 3260^b^
He et al. (2020)	Cardiac surgery (Mixed sex)	10.5 (8.1–12.1)^b^	5650 (4790–7280)^b^
Kammerer et al. (2019)	Cystectomy patients (Mixed sex)	27.9 (14.3–58.8)^b^	1189.3 (998.2–1823.5)

^a^ NR = Not reported.

^b^ Data presented as mean ± SD or median (25th–75th percentile).
